# PLPP/CIN-mediated NF2 S10 dephosphorylation distinctly regulates kainate-induced seizure susceptibility and neuronal death through PAK1-NF-κB-COX-2-PTGES2 signaling pathway

**DOI:** 10.1186/s12974-023-02788-9

**Published:** 2023-04-28

**Authors:** Ji-Eun Kim, Duk-Shin Lee, Tae-Hyun Kim, Hana Park, Min-Ju Kim, Tae-Cheon Kang

**Affiliations:** Department of Anatomy and Neurobiology, Institute of Epilepsy Research, College of Medicine, Hallym University, Chuncheon, Kangwon-Do 24252 South Korea

**Keywords:** COX-2, IPA-3, Merlin, NF-κB, NF2, PAK1, PLPP/CIN, PTGES2

## Abstract

**Background:**

Pyridoxal-5′-phosphate phosphatase/chronophin (PLPP/CIN) selectively dephosphorylates serine (S) 10 site on neurofibromin 2 (NF2, also known as merlin (**m**oesin-**e**zrin-**r**adixin-**l**ike prote**in**) or schwannomin). p21-activated kinase 1 (PAK1) is a serine/threonine protein kinase, which is involved in synaptic activity and plasticity in neurons. NF2 and PAK1 reciprocally regulate each other in a positive feedback manner. Thus, the aim of the present study is to investigate the effects of PLPP/CIN-mediated NF2 S10 dephosphorylation on PAK1-related signaling pathways under physiological and neuroinflammatory conditions, which are largely unknown.

**Methods:**

After kainate (KA) injection in wild-type, *PLPP/CIN*^*−/−*^ and *PLPP/CIN*^*Tg*^ mice, seizure susceptibility, PAK1 S204 autophosphorylation, nuclear factor-κB (NF-κB) p65 S276 phosphorylation, cyclooxygenase-2 (COX-2) upregulation, prostaglandin E synthase 2 (PTGES2) induction and neuronal damage were measured. The effects of 1,1'-dithiodi-2-naphthtol (IPA-3, a selective inhibitor of PAK1) pretreatment on these responses to KA were also validated.

**Results:**

PLPP/CIN overexpression increased PAK1 S204 autophosphorylation concomitant with the enhanced NF2 S10 dephosphorylation in hippocampal neurons under physiological condition. Following KA treatment, PLPP/CIN overexpression delayed the seizure on-set and accelerated PAK1 S204 phosphorylation, NF-κB p65 S276 phosphorylation, COX-2 upregulation and PTGES2 induction, which were ameliorated by PLPP/CIN deletion or IPA-3. Furthermore, IPA-3 pretreatment shortened the latency of seizure on-set without affecting seizure severity (intensity) and ameliorated CA3 neuronal death induced by KA.

**Conclusions:**

These findings indicate that PLPP/CIN may regulate seizure susceptibility (the latency of seizure on-set) and CA3 neuronal death in response to KA through NF2-PAK1-NF-κB-COX-2-PTGES2 signaling pathway.

**Supplementary Information:**

The online version contains supplementary material available at 10.1186/s12974-023-02788-9.

## Introduction

Pyridoxal-5′-phosphate phosphatase/chronophin (PLPP/CIN) is a phosphatase for pyridoxal-5′-phosphate (PLP, an active form of vitamin B_6_) [1]. PLPP/CIN is later identified as an activator for cofilin (a filamentous actin (F-actin) severing protein) by dephosphorylating its serine (S) 3 residue [2–6]. PLPP/CIN also regulates synaptic plasticity and neuronal excitability through F-actin-dependent and -independent pathways [6–11]. Interestingly, seizure activity increases PLPP/CIN expression in hippocampal neurons [3, 11]. Furthermore, PLPP/CIN augments seizure intensity and its progression in response to kainate (KA), while it delays the seizure on-set [7–11]. These roles of PLPP/CIN in seizure activity indicate that the underlying mechanisms of the seizure susceptibility (initiation) and its severity (intensity and progression) may be distinct and complicated, which has been elusive.p21-activated kinase (PAK) family are ubiquitous serine/threonine protein kinases. Among them, PAK1 is first discovered as an effector of Rho GTPases in the rat brain, which is involved in various cellular events such as cytoskeletal dynamics, neurogenesis and apoptosis [12–14]. Since constitutively-active PAK1 rescues N-methyl-D-aspartate receptor (NMDAR)-dependent malformation of dendritic spines [15], PAK1 plays a role in synaptic activity and plasticity in neurons. Thus, the dysregulation or mutation of PAK1 causes mental retardation, Huntington’s disease, Alzheimer’s disease (AD) and epilepsy [16, 17]. PAK1 kinase activity is autoinhibited by homodimerization. Upon GTPase binding via cell division cycle 42 (Cdc42) or Ras-related C3 botulinum toxin substrate 1 (Rac1), PAK1 changes its conformation for autophosphorylation leading to the PAK1 dimer dissociation and its activation [17, 18]. During this process, PAK1 S204 autophosphorylation interferes with the interaction of Src homology 3 (SH3)-polyproline (PxxP) binding proteins, which prevents PAK1 from reverting to an inactive conformation [18, 19].

Neurofibromin 2 (NF2, also known as merlin (**m**oesin-**e**zrin-**r**adixin-**l**ike prote**in**) or schwannomin) is a tumor suppressor protein encoded by the neurofibromatosis type 2 gene *NF2*. Deletion or loss-of-function mutation of *NF2* causes neurofibromatosis type 2, which is a dominant inherited disorder that is characterized by the development of multiple benign tumors of the nervous system. NF2 also affects neuronal excitability by regulating F-actin stability and murine double minute-2 (Mdm2)-mediated postsynaptic density 95 (PSD95) degradation [7, 20]. Furthermore, NF2 and PAK1 reciprocally regulate each other in a positive feedback manner. NF2 S518 phosphorylation by PAK1 or protein kinase A (PKA) leads to conformational changes of NF2 from active (closed) to inactive (open) forms, and inhibits NF2 tumor suppressor activity by blocking its head-to-tail interaction [21, 22]. In turn, this inactive form of NF2 increases PAK1 activity by inhibiting NF2 binding to p21-binding domain on PAK1 [23, 24], which subsequently activates **L**IN-11, **I**sl-1 and **M**EC-3 domain **k**inase 1 (LIMK1) that abrogates F-actin depolymerizing activity of cofilin by S3 phosphorylation [13, 25, 26].

Unlike S518 phosphorylation, PKA-mediated NF2 S10 phosphorylation cannot change its conformation [27]. Furthermore, S10 site on NF2 is dephosphorylated by PLPP/CIN, while S518 site dephosphorylation is mediated by myosin phosphatase-protein phosphatase 1δ (PP1δ) [7, 28]. This PLPP/CIN-mediated NF2 S10 dephosphorylation regulates Mdm2-mediated PSD95 degradations and F-actin stability in an activity-dependent manner [7]. Considering the roles of PAK1 and NF2 in F-actin dynamics and synaptic strength [7, 13, 15, 25, 26], it is noteworthy whether PLPP/CIN-mediated NF S10 dephosphorylation influences PAK1-mediated signaling pathways under physiological and pathological conditions, which is largely unknown.

Here, we demonstrate that PLPP/CIN-mediated NF2 S10 dephosphorylation increased PAK1 S204 autophosphorylation in hippocampal neurons under physiological condition. Following KA treatment, PLPP/CIN-NF2-PAK1 signaling pathway accelerated nuclear factor-κB (NF-κB) p65 S276 phosphorylation, cyclooxygenase-2 (COX-2) upregulation and prostaglandin E synthase 2 (PTGES2) induction. Furthermore, 1,1'-dithiodi-2-naphthtol (IPA-3, a selective inhibitor of PAK1 [29–31]) shortened the latency of seizure on-set, abrogated COX-2 induction and PTGES2 upregulation, and ameliorated CA3 neuronal damage following KA treatment without affecting seizure severity (intensity). Therefore, our findings suggest that PLPP/CIN may distinctly regulate seizure susceptibility (the latency of seizure on-set) and CA3 neuronal death in response to KA through NF2-PAK1-NF-κB-COX-2-PTGES2 signaling pathway.

## Materials and methods

### Experimental animals and chemicals

Male *PLPP/CIN*^*−/−*^ (129/SvEv-C57BL/6 J background) and *PLPP/CIN*^*Tg*^ (C57BL/6J background) mice (8 weeks old) were used in the present study. Wild-type (WT) mice obtained from 129/SvEv-C57BL/6 J and C57BL/6J strains were separately maintained and used as control animals for *PLPP/CIN*^*−/−*^ and *PLPP/CIN*^*Tg*^ mice, respectively. Animals were housed in a standard environment (humidity of 55 ± 5% and a temperature of 22 ± 2 °C on a 12 h light/dark cycle) and provided food and water ad libitum. All animal studies were performed in accordance with protocols approved by the Institutional Animal Care and Use Committee of Hallym University (Chuncheon, South Korea, Code number: #Hallym 2021-3, Approval date: 27th April, 2021). Every effort was made to reduce the number of animals employed and to minimize animal discomfort. All reagents were obtained from Sigma-Aldrich (St. Louis, MO, USA), except as noted.

### Electrode implantation and electroencephalogram (EEG) recording

Animals were anesthetized with Isoflurane (3% induction, 1.5—2% for surgery and 1.5% maintenance in a 65:35 mixture of N_2_O:O_2_). Monopolar electrode (Plastics One, USA) was stereotaxically implanted into the left dorsal hippocampus (2 mm posterior; 1.25 mm lateral; 2 mm depth from bregma). The reference electrode was placed in the posterior cranium over the cerebellum. Three days after surgery when baseline electroencephalogram (EEG) recovers from post-surgery peroxisomal discharges [7–10], EEG in each animal was recorded for 40 min using DAM 80 differential amplifier (0.1–1000 Hz bandpass; World Precision Instruments, USA). Some WT and *PLPP/CIN*^*Tg*^ mice were given IPA-3 (a PAK1 inhibitor, 3.5 mg/kg, i.p. [29–31]) or vehicle 20 min before KA injection. Thereafter, saline or KA (25 mg/kg, i.p.) was administered. This non-lethal KA dose constantly induces seizure activity in both *PLPP/CIN*^*Tg*^ and *PLPP/CIN*^*−/−*^ mice in our previous studies [7–10]. EEG was acquired for 2 h post-KA injection and analyzed using LabChart Pro v7 software (sampling rate: 1000 Hz, AD Instruments, Australia). Electrographic seizures were defined as rhythmic discharges (4–10 Hz) with high amplitude (> two-fold of the baseline EEG). Total EEG power was automatically normalized by the baseline power with LabChart Pro v7 software. Behavioral seizure activity was also evaluated based on the seizure score as followed: (0) no change, (1) no movement, (2) increase in muscle tone at rest, (3) head bobbing/scratching or and circling, (4) clonus/rearing/falling of forelimb, (5) repetitive behavior of 4, (6) severe tonic–clonic seizures [32]. After recording, animals were used for Western blot [7–10].

### Western blot

Animals were decapitated under urethane anesthesia (1.5 g/kg, i.p.). Thereafter, the hippocampi were rapidly dissected and homogenized in lysis buffer. Lysis buffer contained protease inhibitor cocktail (Roche Applied Sciences, Branford, CT, USA) and phosphatase inhibitor cocktail (PhosSTOP®, Roche Applied Science, Branford, CT, USA). Protein concentration was determined using a Micro BCA Protein Assay Kit (Pierce Chemical, Rockford, IL, USA). An equal amount (10 μg) was loaded on a Bis–Tris sodium dodecyl sulfate-poly-acrylamide gel (SDS-PAGE). The proteins were separated by electrophoresis and transferred to membranes. The membranes were blocked with Tris-buffered saline (TBS; in mM 10 Tris, 150 NaCl, pH 7.5, and 0.05% Tween 20) containing 2% bovine serum albumin and then incubated with primary antibodies (Table 1) overnight at 4 °C. The proteins were visualized using electrochemiluminescence (ECL) Western Blotting System (GE Healthcare Korea, Seoul, Korea). For data normalization, β-actin was used as an internal reference. The ratio of phosphoprotein to total protein was described as phosphorylation ratio. ImageQuant LAS4000 system (GE Healthcare Korea, Seoul, South Korea) was used to detect and quantify the Western blot data [7–10].

**Table 1:** Primary antibodies and lectin used in the present study

Antigen	Host	Manufacturer (catalog number)	Dilution used
COX-2	Goat	Abcam (ab23672)	1:1000 (WB) 1:500 (IH)
NeuN	Guinea pig	Millipore (#ABN90P)	1:2000 (IH)
NF2	Rabbit	Elabscience (ENT3080)	1:1000 (WB)
NF2 S10	Rabbit	Signalway antibody (#12334)	1:1000 (WB)
p65-S276	Rabbit	Abcam (ab106129)	1:200 (IH)
PAK1	Rabbit	Novus Biologicals (NBP1-51317)	1:1000 (WB)
PAK1 S204	Rabbit	LifeSpan BioSciences (LS-C357282)	1:1000 (WB) 1:500 (IH)
PLPP/CIN	Rabbit	Sigma (HPA001099)	1:1000 (WB)
PTGES2	Rabbit	Bioss (bs-2639R)	1:1000 (WB) 1:200 (IH)
β-actin	Mouse	Sigma (#A5316)	1:5000 (WB)

*IH* Immunohistochemistry, *WB* Western blot

### Immunohistochemistry and Fluoro-Jade B (FJB) staining

Animals were injected with KA (25 mg/kg, i.p.) or normal saline 20 min after IPA-3 (3.5 mg/kg, i.p.) or vehicle treatment. Two hours after KA injection, animals were perfused with 4% paraformaldehyde through the ascending aorta under deep anesthesia with urethane (1.5 g/kg, i.p.). The brains were then removed and post-fixed in the same fixative for overnight and left in 30% sucrose in phosphate buffer (PB) until sunk. Coronal Sects. (30 μm) were cut using a cryostat. Sections were placed in a plate, rinsed with PBS over 10 min and subsequently blocked for 30 min at room temperature in 10% goat serum (Vector, Burlingame, CA, USA). After blocking, sections were incubated with a cocktail solution containing primary antibodies (Table 1) in PBS containing 0.3% Triton X-100 overnight at room temperature. Sections were then washed over 10 min three times with PBS and incubated with appropriate Cy2- and Cy3-conjugated secondary antibodies for 1 h at room temperature. To establish the specificity of the immunostaining, a negative control test was carried out with pre-immune serum instead of the primary antibody. Because of the highest KA receptor expression, CA3 neurons are the most affected hippocampal neurons in response to KA [33–35]. Thus, the CA3 region is a suitable region to analyze the immunohistochemical data. To measure fluorescent intensity, 5 areas/animals (300 μm^2^/area) were randomly selected in the CA3 region (5 sections from each animal, *n* = 7 in each group). Thereafter, mean intensity on each section was measured by using AxioVision Rel. 4.8 and ImageJ software. Intensity measurements were represented as the number of a 256-Gy scale.

To analyze the KA-induced neuronal damage, we applied Fluoro-Jade B (FJB) staining. Animals used for FJB staining were given diazepam (Valium; Roche, France; 10 mg/kg, i.p.) 2 h after KA injection to control seizure activity. Three days after KA treatment, sections were prepared as the same method as aforementioned. Sections were placed on slides, dried, and immersed in 80% ethanol containing 1% sodium hydroxide. Next, slides were immersed in 70% ethanol solution for 2 min and in purified water for 2 min. After immersion in 0.06% potassium permanganate solution for another 10 min, slides were rinsed with purified water for 2 min. Then, slides were incubated for 30 min in 0.001% FJB (Histo-Chem Inc., USA), freshly prepared by adding 20 ml of a 0.01% stock FJB solution to 180 ml of 0.1% acetic acid, with gentle shaking in the dark. After mounting, areas of interest (the CA3 pyramidal cell layer, 10^4^ μm^2^) were captured, and were selected. Thereafter, cells count was performed using AxioVision Rel. 4.8 software [7–10].

### Data analysis

After evaluating the values on normality using Shapiro–Wilk *W*-test, Student *t*-test, repeated measured ANOVA, Friedman test or two-way analysis of variance (ANOVA) followed by Newman-Keuls *post-hoc* test was used to analyze statistical significance. Linear regression analysis (correlation test) was also performed using data obtained from the same animal. A *p* < 0.05 was considered to be statistically different [7–10].

## Results

### PLPP/CIN-mediated NF2 S10 dephosphorylation increases PAK1 activity under physiological condition

**Fig. 1:**
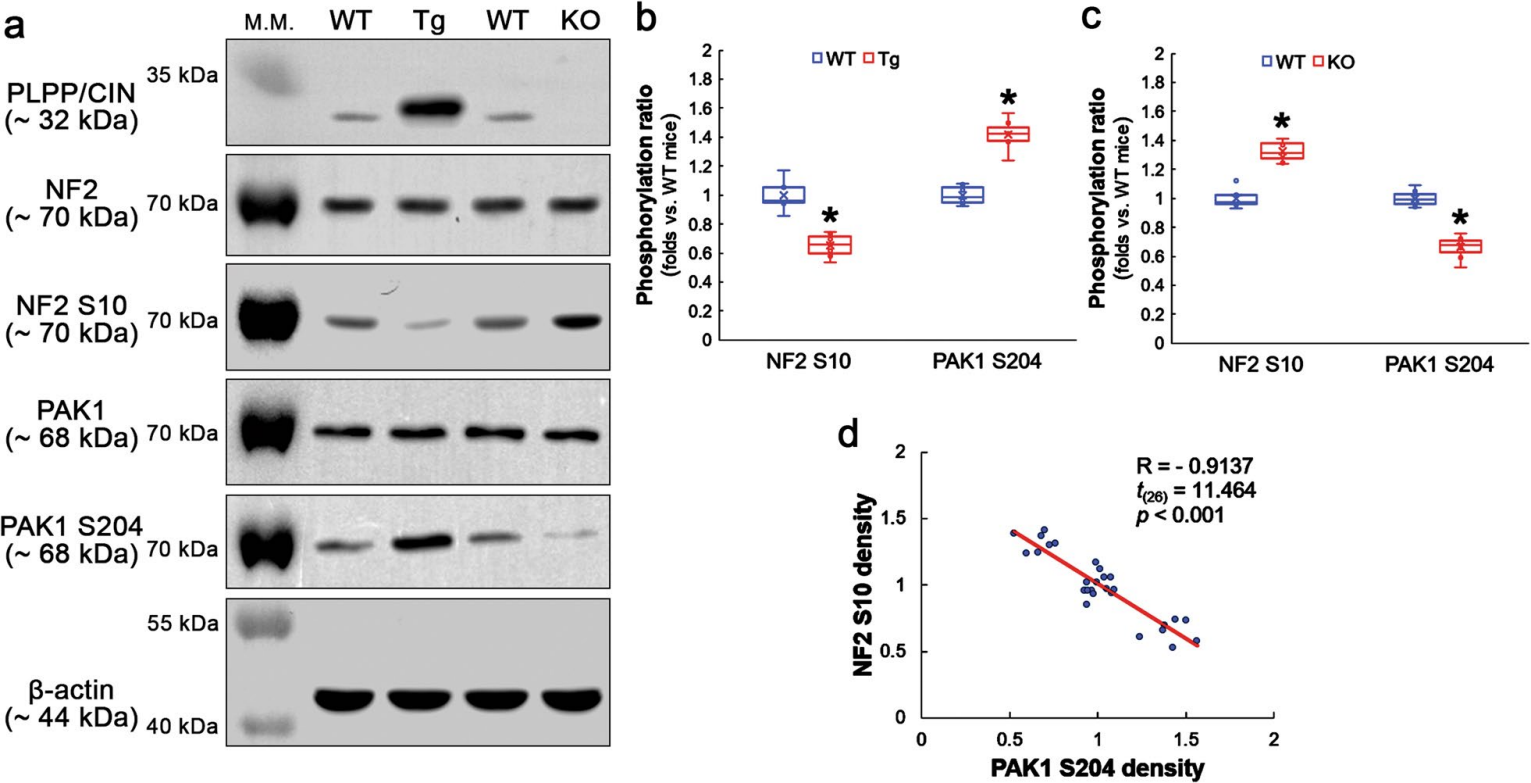
Effects of PLPP/CIN overexpression and its deletion on phosphorylations of NF2 and PAK1 under physiological condition. PLPP/CIN overexpression reduces NF2 S10 phosphorylation in a coupled with increased PAK1 S204 phosphorylation, which are reversed by its deletion. **a** Representative Western blot data of phosphorylations of NF2 and PAK1 in the whole hippocampus. **b**, **c** Quantifications of NF2 S10 and PAK1 S204 phosphorylations based on Western blot data (*p < 0.05 vs. WT animals, *n* = 7, respectively). **d** Linear regression analysis between NF2 S10 and PAK1 S204 phosphorylations

Recently, we have reported that PLPP/CIN dephosphorylates NF2 S10 site without altering its S518 phosphorylation [7]. NF2-mediated suppresses PAK1 activity, and S204 site in PAK1 is the primary autophosphorylation site to control enzymatic activity [18, 36, 37]. Therefore, we investigated whether PLPP/CIN-mediated NF2 S10 dephosphorylation affects PAK1 S204 phosphorylation level under physiological condition. In the present study, NF2 S10 phosphorylation level in *PLPP/CIN*^*Tg*^ mice was 0.65-fold of WT mice level under physiological condition (*t*_(12)_ = 7.07, *p* < 0.001 vs. WT mice, Student’s *t*-test, *n* = 7, respectively; Fig. 1a, b and Additional file 1: Fig. S1), while its phosphorylation level in *PLPP/CIN*^*−/−*^ mice was 1.32-fold higher than that in WT mice (*t*_(12)_ = 9.14, *p* < 0.001 vs. WT mice, Student’s *t*-test, *n* = 7, respectively; Fig. 1a, c and Additional file 1: Fig. S1). In contrast to NF2 S10 phosphorylation, PAK1 S204 autophosphorylation level in *PLPP/CIN*^*Tg*^ mice was 1.42-fold of WT mice level (*t*_(12)_ = 9.07, *p* < 0.001 vs. WT mice, Student’s *t*-test, *n* = 7, respectively; Fig. 1a–c and Additional file 1: Fig. S1), while its level in *PLPP/CIN*^*−/−*^ mice was 0.66-fold of WT mice level (*t*_(12)_ = 9.14, *p* < 0.001 vs. WT mice, Student’s *t*-test, *n* = 7, respectively; Fig. 1a, c and Additional file 1: Fig. S1). Linear regression analysis demonstrated an inverse relationship between NF2 S10 and PAK1 S204 phosphorylations (*R* =—0.91, *t*_(26)_ = 11.46, *p* < 0.001, *n* = 7, respectively; Fig. 1d). These findings indicate that PLPP/CIN-mediated NF2 S10 dephosphorylation may increase PAK1 S204 autophosphorylation under physiological condition.

### KA increases PLPP/CIN-mediated NF2 S10 dephosphorylation and PAK1 S204 phosphorylation

**Fig. 2:**
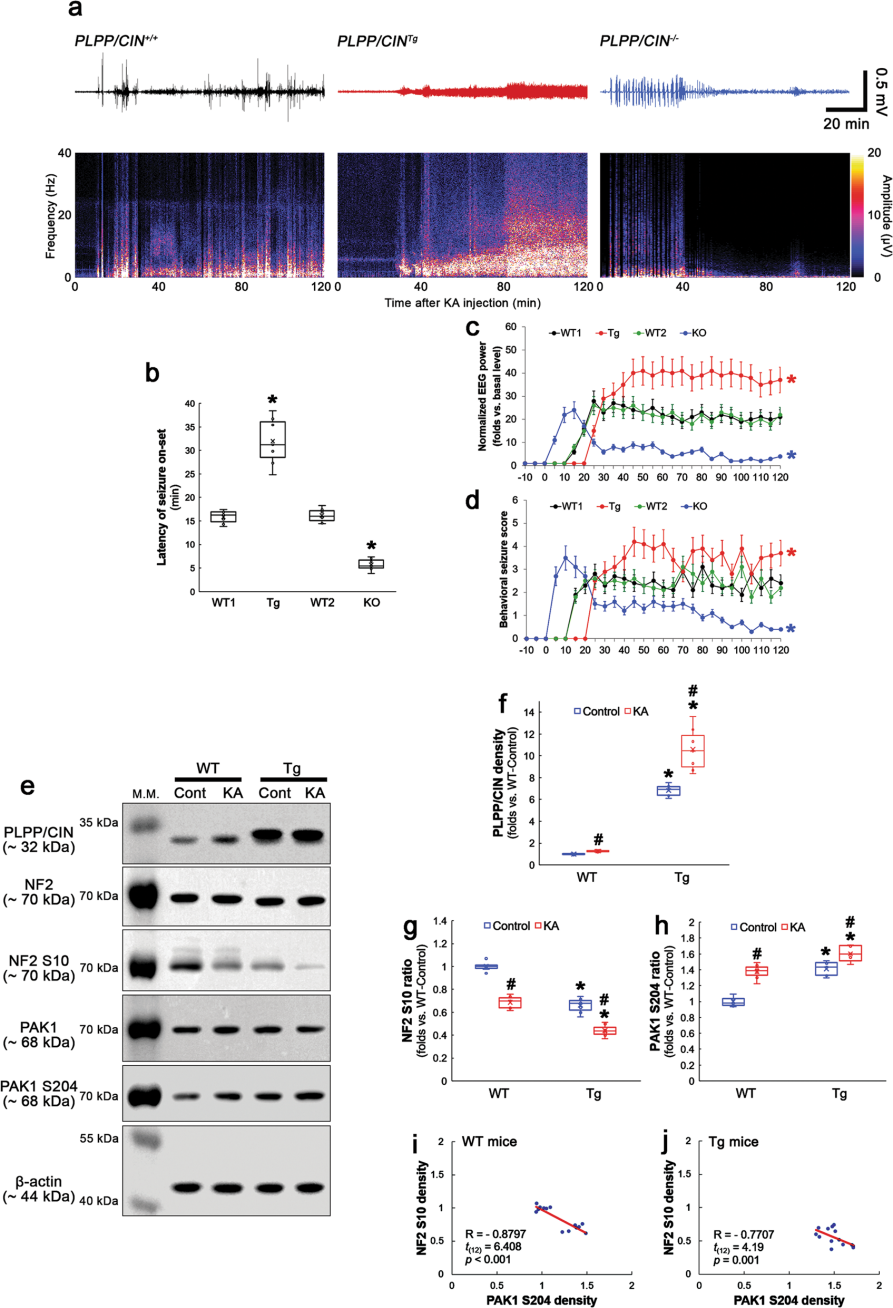
Effects of PLPP/CIN overexpression on phosphorylations of NF2 and PAK1 following KA injection. PLPP/CIN overexpression delays seizure on-set and increases its intensity in response to KA, which are which are reversed by its deletion. Two hours after KA injection, PLPP/CIN overexpression reduces NF2 S10 phosphorylation in a coupled with enhanced PAK1 S204 phosphorylation. **a** Representative EEG (upper panels) and frequency-power spectral temporal mans (lower panels) in response to KA in *PLPP/CIN*^+*/*+^, *PLPP/CIN*^*Tg*^ and *PLPP/CIN*^*−/−*^ mice. **b**–**d** Quantitative analyses of the latency of seizure on-set, total EEG power and behavioral seizure severity in response to KA (*p < 0.05 vs. WT mice; *n* = 7, respectively). **e** Representative Western blot data of phosphorylations of NF2 and PAK1 in the whole hippocampus. **f**–**h** Quantifications of PLPP/CIN expression, NF2 S10 and PAK1 S204 phosphorylations based on Western blot data (*p < 0.05 vs. WT animals and ^#^*p* < 0.05 vs. control animals with Newman-Keuls *post-hoc* test, *n* = 7, respectively). (**i**–**j**) Linear regression analyses between NF2 S10 and PAK1 S204 phosphorylations in WT and *PLPP/CIN*^*Tg*^ mice

To further explore the role of PLPP/CIN-mediated NF2 S10 dephosphorylation in PAK1 activity, we investigated whether the altered NF2 S10 phosphorylation is relevant to the changed PAK1 S204 phosphorylation following KA injection. Consistent with our previous studies [7–10], *PLPP/CIN*^*Tg*^ mice showed the longer latency of seizure on-set (*t*_(12)_ = 8.1, *p* < 0.001, Student’s *t*-test, *n* = 7, respectively; Fig. 2a, b) and the higher seizure intensity (severity) in response to KA (*F*_(1,12)_ = 9.53, *p* = 0.009, repeated measure one-way ANOVA, *n* = 7, respectively; Fig. 2a and c), as compared to WT mice. Consistent with the seizure intensity, PLPP/CIN overexpression aggravated behavioral seizure activity (*χ*^*2*^_(1)_ = 10.07, *p* = 0.018, Friedman test, *n* = 7, respectively; Fig. 2d). In contrast, *PLPP/CIN*^*−/−*^ mice showed the decrease in the latency of seizure on-set (*t*_(12)_ = 14.22, *p* < 0.001, Student’s *t*-test, *n* = 7, respectively; Fig. 2a, b) and seizure intensity/duration in response to KA (*F*_(1,12)_ = 11.27, *p* = 0.006, repeated measure one-way ANOVA, *n* = 7, respectively; Fig. 2a and c). PLPP/CIN deletion also attenuated behavioral seizure activity (*χ*^*2*^_(1)_ = 12.45, *p* = 0.006, Friedman test, *n* = 7, respectively; Fig. 2d).

Compatible with our previous studies [7, 11], KA increased PLPP/CIN expression in WT and *PLPP/CIN*^*Tg*^ mice, respectively (*F*_*group*(1,24)_ = 386.08, *p* < 0.001; *F*_*KA*(1,24)_ = 27.41, *p* < 0.001; *F*_group*KA(1,24)_ = 20.67, *p* < 0.001; two-way ANOVA, *n* = 7, respectively; Fig. 2e, f and Additional file 1: Fig. S2). In addition, KA decreased NF2 S10 phosphorylation level to 0.69- and 0.44-fold of control WT mice level in WT and *PLPP/CIN*^*Tg*^ mice, respectively (*F*_*group*(1,24)_ = 216.81, *p* < 0.001; *F*_*KA*(1,24)_ = 179.74, *p* < 0.001; *F*_group*KA(1,24)_ = 5.43, *p* = 0.029; two-way ANOVA, *n* = 7, respectively; Fig. 2e, g and Additional file 1: Fig. S2). In contrast, KA increased PAK1 S204 phosphorylation level to 1.38- and 1.6-fold of control WT mice level in WT and *PLPP/CIN*^*Tg*^ mice, respectively (*F*_*group*(1,24)_ = 71.35, *p* < 0.001; *F*_*KA*(1,24)_ = 7.69, *p* < 0.001; *F*_group*KA(1,24)_ = 90.04, *p* = 0.011; two-way ANOVA, *n* = 7, respectively; Fig. 2e, h and Additional file 1: Fig. S2). Linear regression analysis revealed inverse relationships between NF2 S10 and PAK1 S204 phosphorylations in WT (*R* = − 0.88, *t*_(12)_ = 6.408, *p* < 0.001, *n* = 7, respectively; Fig. 2i) and *PLPP/CIN*^*Tg*^ mice (*R* =—0.771, *t*_(12)_ = 4.19, *p* = 0.001, *n* = 7, respectively; Fig. 2j).

**Fig. 3:**
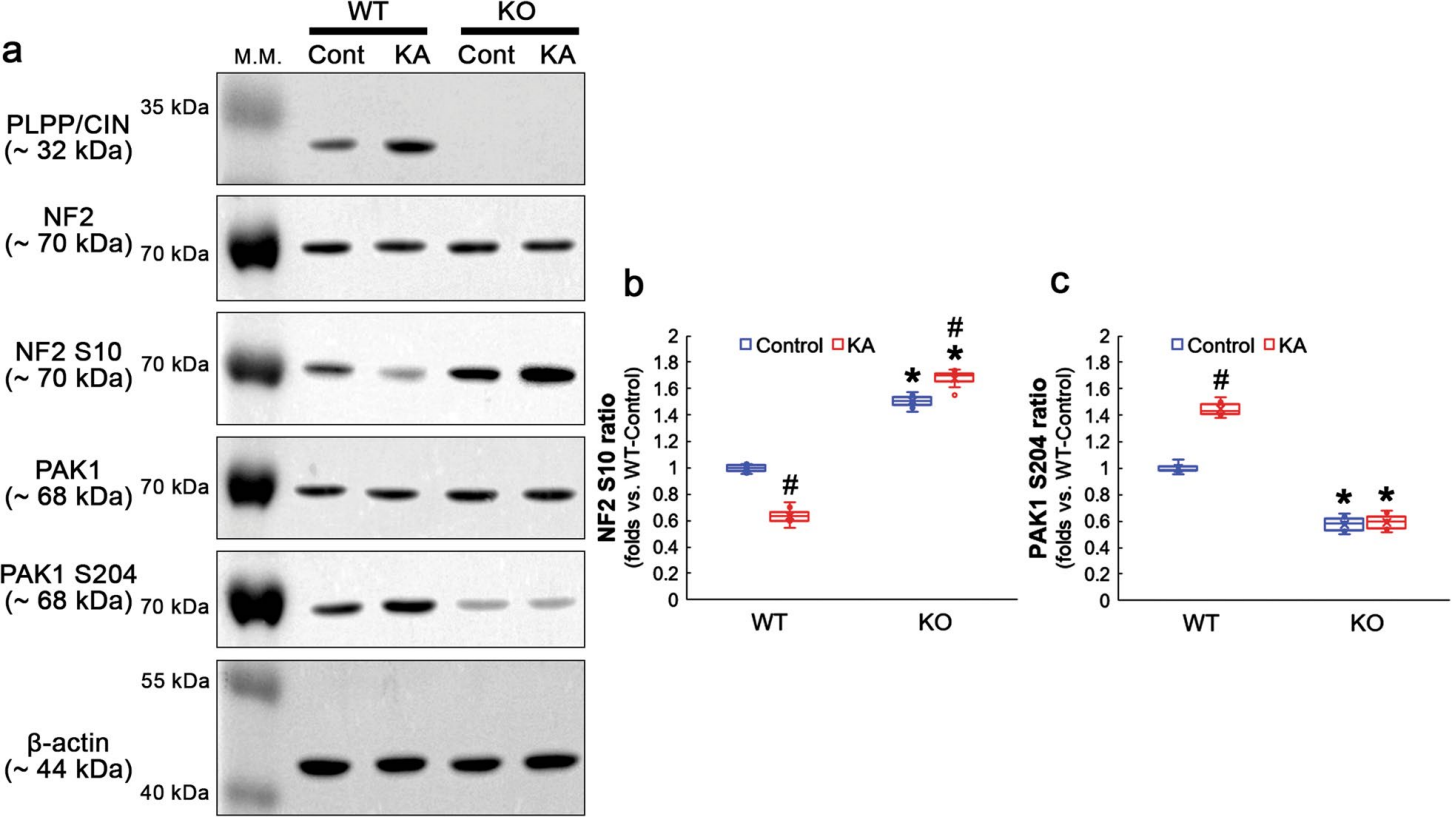
Effects of PLPP/CIN ablation on phosphorylations of NF2 and PAK1 following KA injection. Two hours after KA injection, PLPP/CIN ablation increases NF2 S10 phosphorylation without affecting PAK1 S204 phosphorylation. **a** Representative Western blot data of phosphorylations of NF2 and PAK1 in the whole hippocampus. **b**, **c** Quantifications of NF2 S10 and PAK1 S204 phosphorylations based on Western blot data (*p < 0.05 vs. WT animals and ^#^*p* < 0.05 vs. control animals with Newman-Keuls *post-hoc* test, *n* = 7, respectively)

In *PLPP/CIN*^*−/−*^ mice, KA increased NF2 S10 phosphorylation level to 1.7-fold of control WT mice level (*F*_*group*(1,24)_ = 1315.08, p < 0.001; *F*_*KA*(1,24)_ = 20.49, p < 0.001; *F*_group*KA(1,24)_ = 158.23, *p* < 0.001; two-way ANOVA, *n* = 7, respectively; Fig. 3a, b and Additional file 1: Fig. S3). However, KA did not affect PAK1 S204 phosphorylation level in *PLPP/CIN*^*−/−*^ mice (*F*_*group*(1,24)_ = 971.14, p < 0.001; *F*_*KA*(1,24)_ = 128.85, p < 0.001; *F*_group*KA(1,24)_ = 109.03, *p* < 0.001; two-way ANOVA, *n* = 7, respectively; Fig. 3a, c and Additional file 1: Fig. S3). These findings indicate that PLPP/CIN-mediated NF2 S10 dephosphorylation may affect KA-induced seizure activity by regulating PAK1 S204 autophosphorylation.

### PAK1 inhibition shortens the latency of seizure on-set without altering seizure duration and its progression in response to KA

**Fig. 4:**
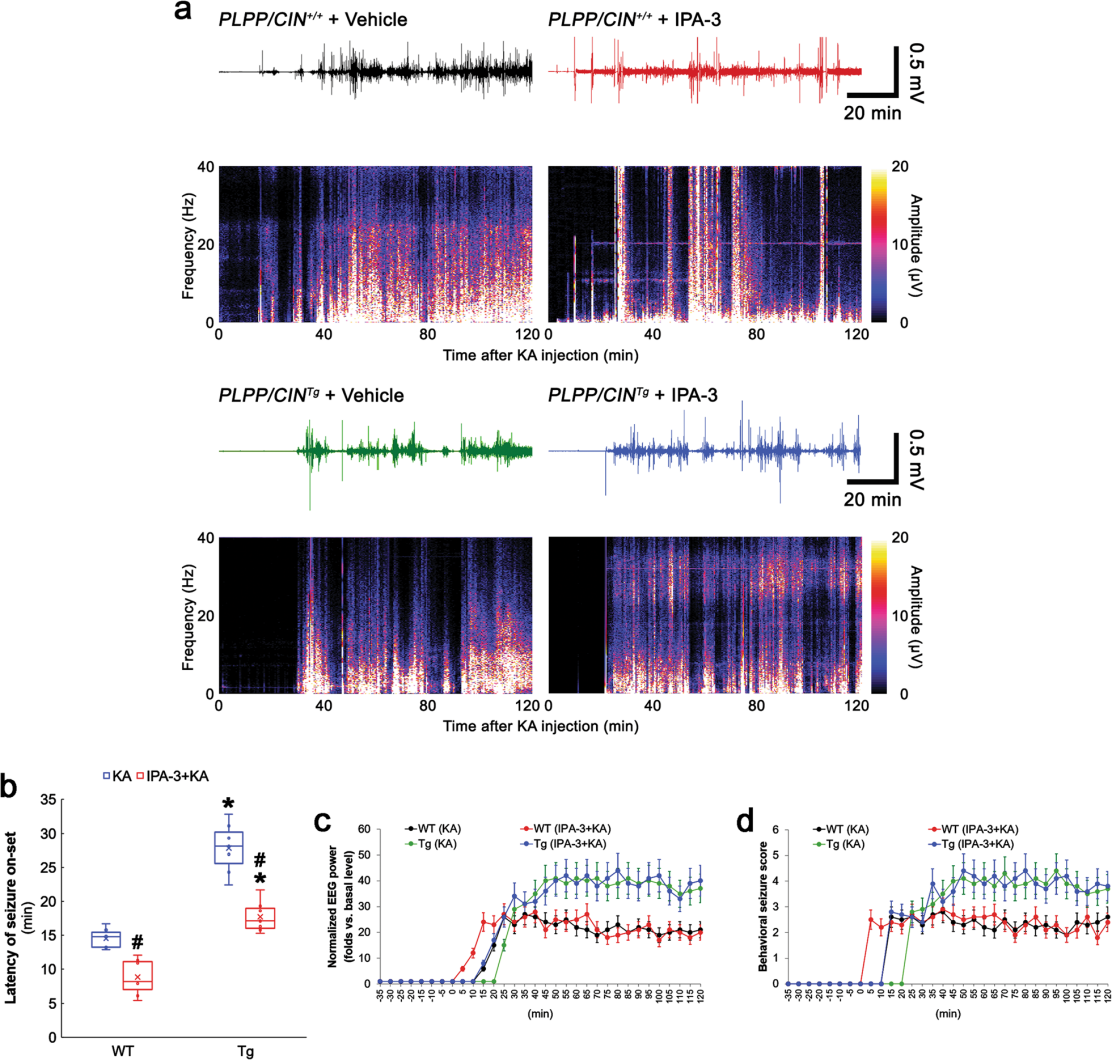
Effects of IPA-3 pretreatment on KA-induced seizure activity in *PLPP/CIN*^+*/*+^ and *PLPP/CIN*^*Tg*^ mice. IPA-3 pretreatment shortens seizure on-set in response to KA in *PLPP/CIN*^+*/*+^ and *PLPP/CIN*^*Tg*^ mice, without affecting its intensity. **a** Representative EEG (upper panels) and frequency-power spectral temporal mans (lower panels) in response to KA in *PLPP/CIN*^+*/*+^and *PLPP/CIN*^*Tg*^ mice. **b**–**d** Quantitative analyses of the latency of seizure on-set, total EEG power and behavioral seizure severity in response to KA (*p < 0.05 vs. WT mice and ^#^*p* < 0.05 vs. KA-treated animals with Newman-Keuls *post-hoc* test, *n* = 7, respectively)

To confirm the role of PAK1 S204 phosphorylation in KA-induced seizure activity, we applied IPA-3 (a PAK1 inhibitor, 3.5 mg/kg, i.p.), which is permeable to the brain-blood barrier [29–31], in WT and *PLPP/CIN*^*Tg*^ mice 20 min prior to KA injection. Although IPA-3 pretreatment did not affect the basal EEG level, it decreased the latency of seizure on-set in response to KA from 14.5 to 8.8 min in WT mice and from 27.8 to 17.7 min in *PLPP/CIN*^*Tg*^ mice, respectively (*F*_*group*(1,24)_ = 124.76, p < 0.001; *F*_*treatment*(1,24)_ = 63.39, p < 0.001; *F*_group*treatment(1,24)_ = 4.94, *p* = 0.036; two-way ANOVA, *n* = 7, respectively; Fig. 4a, b). However, IPA-3 did not influence seizure intensity and behavioral seizure severity in response to KA in WT and *PLPP/CIN*^*Tg*^ mice (Fig. 4a–d). These findings indicate that PLPP/CIN-mediated PAK1 activation may be involved in the delay of seizure on-set in response to KA.

### PLPP/CIN-mediated PAK1 S204 phosphorylation rapidly induces neuronal NF-κB transactivation following KA injection

NF-κB is involved in the regulation of seizure activity and postictal events. Briefly, KA acutely activates NF-κB in hippocampal neurons [38], and NF-κB inhibition decreases the latencies of on-set of seizures and status epilepticus in response to KA [39]. Interestingly, PAK1 is required for the promotion of NF-κB activation by multiple stimuli [40]. Furthermore, NF2 inhibits PAK1-mediated NF-κB activation [36, 37]. Since phosphorylation is essential for optimal NF-κB activation [41] and KA initially induces epileptiform discharges in CA3 neurons with subsequent propagation to other hippocampal neurons due to the highest KA receptor [33–35], we examined the alterations in NF-κB p65 S276 phosphorylation in CA3 neurons following KA injection.

**Fig. 5:**
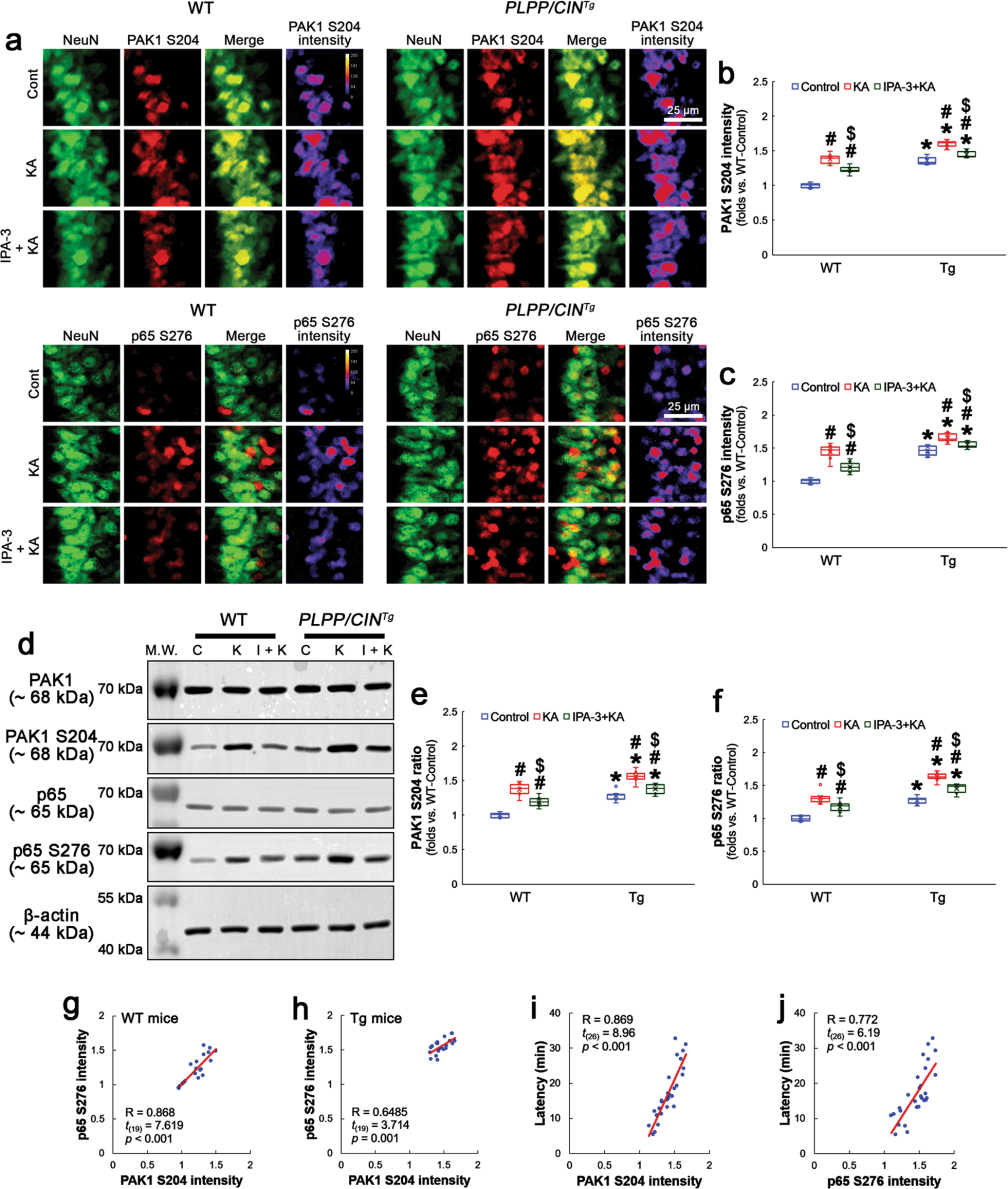
Effects of IPA-3 (a PAK1 inhibitor) on neuronal PAK1 S204 and NF-κB p65 S276 phosphorylations in WT and *PLPP/CIN*^*Tg*^ mice 2 h after KA injection. Under physiological condition, PLPP/CIN overexpression enhances PAK1 S204 and NF-κB p65 S276 phosphorylation levels. As compared to control animals, KA augments PAK1 S204 and p65 S276 phosphorylations in CA3 neurons in WT mice. PLPP/CIN overexpression further increases them. IPA-3 pretreatment ameliorates KA-induced PAK1 S204 and p65 S276 phosphorylations in both strains. **a** Representative photos for PAK1 S204 and p65 S276 phosphorylations in CA3 neurons. **b**, **c** Quantification of PAK1 S204 and p65 S276 phosphorylations based on immunofluorescent data (*p < 0.05 vs. WT mice, ^#^*p* < 0.05 vs. control animals and ^$^*p* < 0.05 vs. KA-treated animals with Newman-Keuls *post-hoc* test, *n* = 7, respectively, *n* = 7, respectively). **d** Representative Western blot data of phosphorylations of PAK1 and p65 in the whole hippocampus. (**e**–**f**) Quantifications of PAK1 S204 and p65 S276 phosphorylations based on Western blot data (*p < 0.05 vs. WT mice, ^#^*p* < 0.05 vs. control animals and ^$^*p* < 0.05 vs. KA-treated animals with Newman-Keuls *post-hoc* test, *n* = 7, respectively). **g**–**h** Linear regression analyses between PAK1 S204 and p65 S276 phosphorylation in WT and *PLPP/CIN*^*Tg*^ mice. **i** Linear regression analysis between PAK1 S204 phosphorylation and the latency of seizure on-set following KA injection. **j** Linear regression analysis between p65 S276 phosphorylation and the latency of seizure on-set following KA injection

Under physiological condition, PAK1 S204 phosphorylation level in CA3 neurons of *PLPP/CIN*^*Tg*^ mice was 1.36-fold of WT mice level. KA increased PAK1 S204 phosphorylation level to 1.38- and 1.6-fold of control WT mice level in WT and *PLPP/CIN*^*Tg*^ mice, respectively (*F*_*group*(1,24)_ = 172.73, p < 0.001; *F*_*KA*(1,24)_ = 198.81, p < 0.001; *F*_group*KA(1,24)_ = 10.24, *p* = 0.004; two-way ANOVA, *n* = 7, respectively; Fig. 5a-b). IPA-3 decreased PAK1 S204 phosphorylation level to 1.23- and 1.45-fold of control WT mice level in WT and *PLPP/CIN*^*Tg*^ mice following KA treatment, respectively (*F*_*group*(1,36)_ = 231.56, p < 0.001; *F*_*treatment*(2,36)_ = 104.46, p < 0.001; *F*_group*treatment(2,36)_ = 7.02, *p* = 0.003; two-way ANOVA, *n* = 7, respectively; Fig. 5a, b). p65 S276 phosphorylation level in *PLPP/CIN*^*Tg*^ mice was 1.46-fold of WT mice level under physiological condition. KA increased p65 S276 phosphorylation level to 1.44- and 1.65-fold of control WT mice level in WT and *PLPP/CIN*^*Tg*^ mice, respectively (*F*_*group*(1,24)_ = 117, p < 0.001; *F*_*KA*(1,24)_ = 104.11, p < 0.001; *F*_group*KA(1,24)_ = 15.5, *p* < 0.001; two-way ANOVA, *n* = 7, respectively; Fig. 5a and c). IPA-3 reduced p65 S276 phosphorylation level to 1.21- and 1.55-fold of control WT mice level in WT and *PLPP/CIN*^*Tg*^ mice following KA treatment, respectively (*F*_*group*(1,36)_ = 193.59, p < 0.001; *F*_*treatment*(2,36)_ = 57.29, p < 0.001; *F*_group*treatment(2,36)_ = 8.52, *p* < 0.001; two-way ANOVA, *n* = 7, respectively; Fig. 5a and c).

Western blot also revealed that KA increased PAK1 S204 phosphorylation level following KA treatment, which was attenuated IPA-3 in both WT and *PLPP/CIN*^*Tg*^ mice (*F*_*group*(1,36)_ = 88.16, p < 0.001; *F*_*treatment*(2,36)_ = 65.51, p < 0.001; *F*_group*treatment(2,36)_ = 1.78, *p* = 0.184; two-way ANOVA, *n* = 7, respectively; Fig. 5d, e and Additional file 1: Fig. S4). Furthermore, IPA-3 attenuated the KA-induced p65 S276 phosphorylation in both WT and *PLPP/CIN*^*Tg*^ mice following KA injection (*F*_*group*(1,36)_ = 153.12, p < 0.001; *F*_*treatment*(2,36)_ = 70.1, p < 0.001; *F*_group*treatment(2,36)_ = 0.38, *p* = 0.686; two-way ANOVA, *n* = 7, respectively; Fig. 5d, f and Additional file 1: Fig. S4).

Linear regression analysis revealed direct proportions between PAK1 S204 and p65 S276 phosphorylations in WT (*R* = 0.868, *t*_(19)_ = 7.619, *p* < 0.001, *n* = 7, respectively; Fig. 5g) and *PLPP/CIN*^*Tg*^ mice (*R* = 0.649, *t*_(19)_ = 3.714, *p* = 0.001, *n* = 7, respectively; Fig. 5h). Furthermore, the latency of seizure on-set showed a direct relationship with PAK1 S204 (*R* = 0.869, *t*_(26)_ = 8.96, p < 0.001, *n* = 7, respectively; Fig. 5i) and p65 S276 (*R* = 0.772, *t*_(26)_ = 6.19, p < 0.001, *n* = 7, respectively; Fig. 5j). These findings indicate that PLPP/CIN-NF2-mediated PAK1 phosphorylation may delay seizure on-set in response to KA by increasing NF-κB activity.

### PLPP/CIN-mediated PAK1 S204 phosphorylation enhances COX-2 and PTGES2 expressions induced by KA

COX-2 is the inducible cyclooxygenase isoform that plays a central role in the inflammatory cascade [42]. COX-2 is upregulated in the hippocampus by KA treatment [43]. COX-2 upregulation activates prostaglandin E2 (PGE_2_) synthesis, which is catalyzed by PTGES2 in neurons under pathophysiological condition [44–46]. Interestingly, seizures elicited by NMDA are more severe in *COX-2*^*−/−*^ mice and COX-2 inhibitors exacerbate seizure activity induced by KA due to the reduced γ-aminobutyric acid type A (GABA_A_) receptor-mediated inhibitions [47–53]. Since NF-κB is an upstream transcription factor of COX-2 during inflammatory processes [54, 55], it is likely that KA-induced COX-2 induction may be regulated by PLPP/CIN-PAK1-NF-κB signaling pathway, which would inhibit the seizure activity in response to KA.

**Fig. 6:**
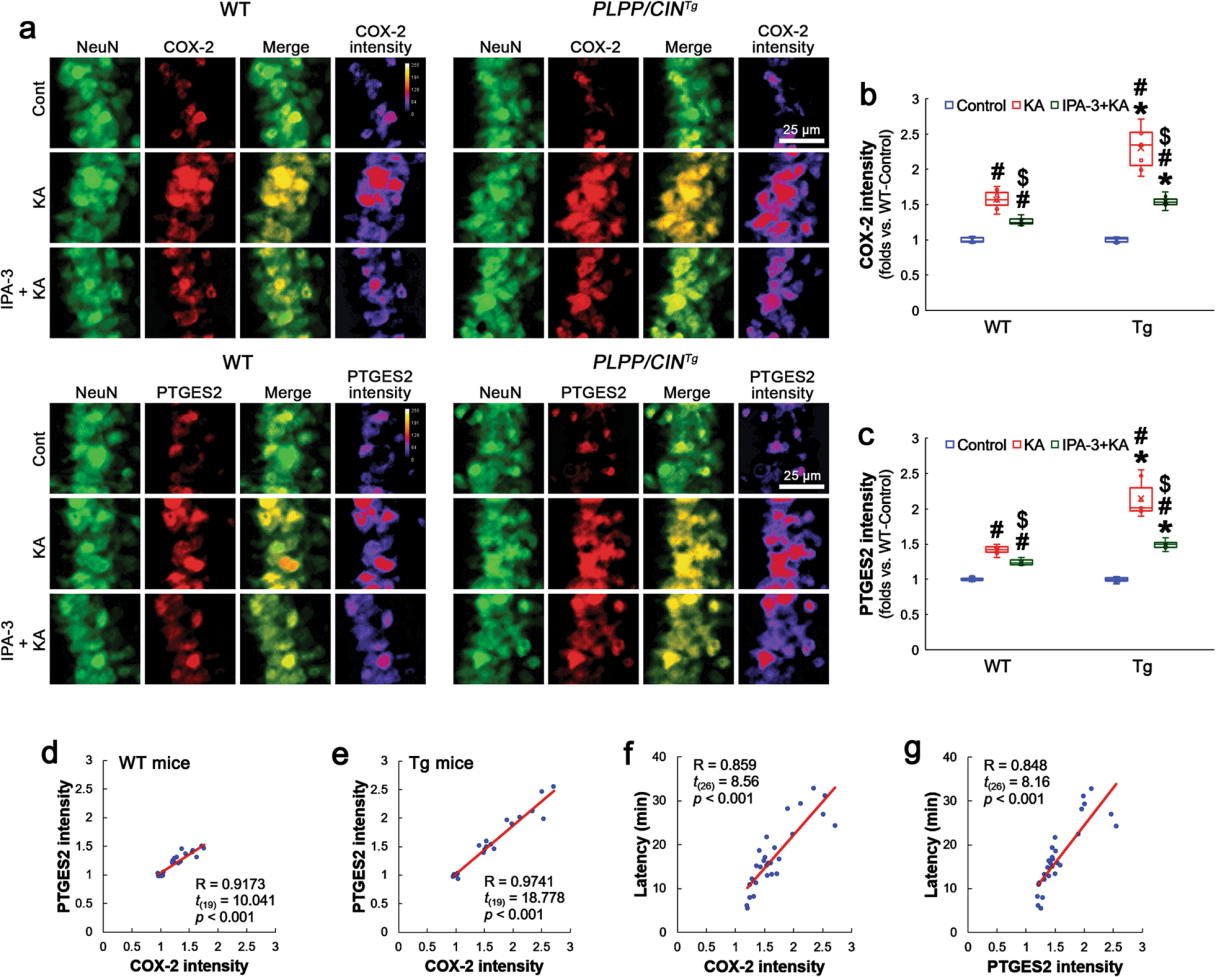
Effects of IPA-3 (a PAK1 inhibitor) on neuronal COX-2 and PTGES2 expression in WT and *PLPP/CIN*^*Tg*^ mice 2 h after KA injection. Under physiological condition, PLPP/CIN overexpression does not alter COX-2 and PTGES2 expression levels. As compared to control animals, KA leads to COX-2 and PTGES2 upregulations in CA3 neurons in WT mice. PLPP/CIN overexpression further augments them. IPA-3 pretreatment attenuates KA-induced COX-2 and PTGES2 inductions in both strains. **a** Representative photos for COX-2 and PTGES2 expressions in CA3 neurons. (**b**–**c**) Quantification of COX-2 and PTGES2 expressions based on immunofluorescent data (*p < 0.05 vs. WT mice, ^#^*p* < 0.05 vs. control animals and ^$^*p* < 0.05 vs. KA-treated animals with Newman-Keuls *post-hoc* test, *n* = 7, respectively). **d**, **e** Linear regression analyses between COX-2 and PTGES2 expressions in WT and *PLPP/CIN*^*Tg*^ mice. **f** Linear regression analysis between COX-2 expression and the latency of seizure on-set following KA injection. **g** Linear regression analysis between PTGES2 expression and the latency of seizure on-set following KA injection

Under physiological condition, there was no difference in COX-2 expression between WT and *PLPP/CIN*^*Tg*^ mice (Fig. 6a, b). KA increased COX-2 level to 1.55- and 1.9-fold of control WT mice level in WT and *PLPP/CIN*^*Tg*^ mice, respectively (*F*_*group*(1,24)_ = 32.05, p < 0.001; *F*_*KA*(1,24)_ = 210.02, p < 0.001; *F*_group*KA(1,24)_ = 31.64, *p* < 0.001; two-way ANOVA, *n* = 7, respectively; Fig. 6a, b). IPA-3 attenuated COX-2 level to 1.27- and 1.54-fold of control WT mice level in WT and *PLPP/CIN*^*Tg*^ mice following KA treatment, respectively (*F*_*group*(1,36)_ = 55.8, p < 0.001; *F*_*treatment*(2,36)_ = 145.76, p < 0.001; *F*_group*treatment(2,36)_ = 22.26, *p* < 0.001; two-way ANOVA, *n* = 7, respectively; Fig. 6a, b). Similar to the case of COX-2, PLPP/CIN overexpression did not affect PTGES2 expression under physiological condition (Fig. 6a and c). KA increased PTGES2 level to 1.42- and 2.14-fold of control WT mice level in WT and *PLPP/CIN*^*Tg*^ mice, respectively (*F*_*group*(1,24)_ = 49.5, p < 0.001; *F*_*KA*(1,24)_ = 234.74, p < 0.001; *F*_group*KA(1,24)_ = 51.27, *p* < 0.001; two-way ANOVA, *n* = 7, respectively; Fig. 6a and c). IPA-3 attenuated PTGES2 level to 1.24- and 1.49-fold of control WT mice level in WT and *PLPP/CIN*^*Tg*^ mice following KA treatment, respectively (*F*_*group*(1,36)_ = 82.56, p < 0.001; *F*_*treatment*(2,36)_ = 162.64, *p* < 0.001; *F*_group*treatment(2,36)_ = 36.6, *p* < 0.001; two-way ANOVA, *n* = 7, respectively; Fig. 6a and c). Thus, direct proportions between COX-2 and PTGES2 expressions were observed in WT (*R* = 0.917, *t*_(19)_ = 10.041, *p* < 0.001, *n* = 7, respectively; Fig. 6d) and *PLPP/CIN*^*Tg*^ mice (*R* = 0.974, *t*_(19)_ = 18.778, *p* < 0.001, *n* = 7, respectively; Fig. 6e). The latency of seizure on-set demonstrated a direct relationship with COX-2 (*R* = 0.859, *t*_(26)_ = 8.56, p < 0.001, *n* = 7, respectively; Fig. 6f) and PTGES2 (*R* = 0.848, *t*_(26)_ = 8.16, p < 0.001, *n* = 7, respectively; Fig. 6g).

**Fig. 7:**
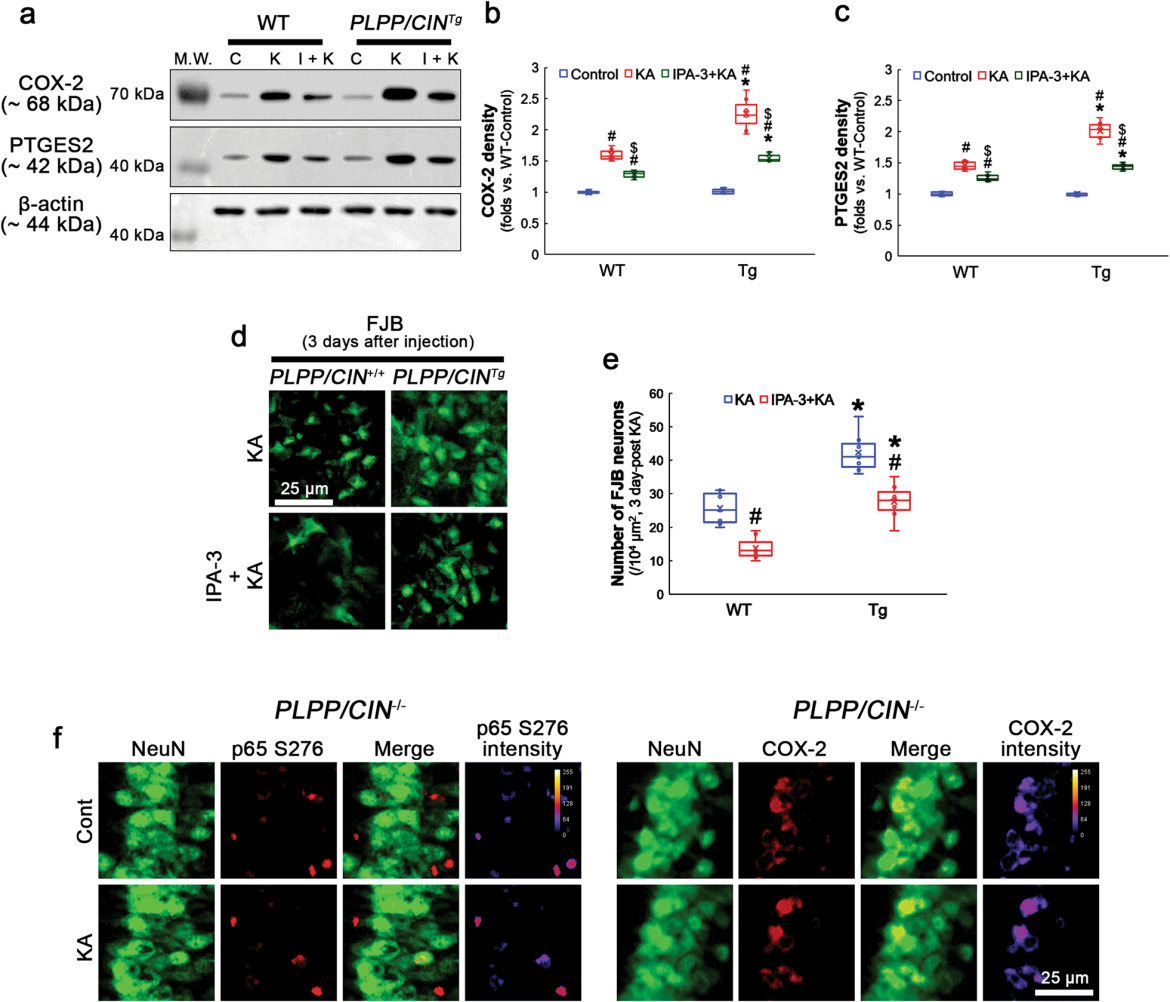
Effects of IPA-3 (a PAK1 inhibitor) on COX-2 and PTGES2 protein levels and KA-induced CA3 neuronal damage in WT and *PLPP/CIN*^*Tg*^ mice and effects of KA on p65 S276 and COX-2 level in *PLPP/CIN*^*−/−*^ mice. PLPP/CIN overexpression enhances KA-induces COX-2 and PTGES2 upregulations and aggravates CA3 neuronal damage 3 days after KA injection, which are ameliorated by IPA-3 pretreatment. PLPP/CIN ablation does not influence KA-induced p65 S276 phosphorylation and COX-2 expression. **a** Representative Western blot data of COX-2 and PTGES2 in the whole hippocampi of WT and *PLPP/CIN*^*Tg*^ mice **b**, **c** Quantifications of COX-2 and PTGES2 levels based on Western blot data (*p < 0.05 vs. WT mice, ^#^*p* < 0.05 vs. control animals and ^$^*p* < 0.05 vs. KA-treated animals with Newman-Keuls *post-hoc* test, *n* = 7, respectively). **d** Representative images for FJB-positive degenerating CA3 neurons at 3 days after KA injection in WT and *PLPP/CIN*^*Tg*^ mice. **e** Quantitation of the number of FJB-positive CA3 neurons (*p < 0.05 vs. WT mice and ^#^*p* < 0.05 vs. KA-treated animals with Newman-Keuls *post-hoc* test, *n* = 7, respectively). **f** Representative photos for p65 S276 phosphorylation and COX-2 expressions in CA3 neurons of *PLPP/CIN*^*−/−*^ mice 2 h after KA injection

Western blot data also revealed that IPA-3 ameliorated KA-induced upregulations of COX-2 (*F*_*group*(1,36)_ = 73.63, *p* < 0.001; *F*_*treatment*(2,36)_ = 216.43, *p* < 0.001; *F*_group*treatment(2,36)_ = 26.84, *p* < 0.001; two-way ANOVA, *n* = 7, respectively; Fig. 7a, b and Additional file 1: Fig. S5) and PTGES2 in both strains (*F*_*group*(1,36)_ = 112.14, *p* < 0.001; *F*_*treatment*(2,36)_ = 338.92, p < 0.001; *F*_group*treatment(2,36)_ = 53.75, *p* < 0.001; two-way ANOVA, *n* = 7, respectively; Fig. 7a, c and Additional file 1: Fig. S5). Compatible with our previous studies [7, 11], CA3 neuronal damage were more severe in *PLPP/CIN*^*Tg*^ mice than WT mice 3 days after KA injection, which were attenuated by IPA-3 pretreatment (*F*_*group*(1,24)_ = 67.18, p < 0.001; *F*_*treatment*(1,24)_ = 50.75, *p* < 0.001; *F*_group*treatment(1,24)_ = 0.59, *p* = 0.451; two-way ANOVA, *n* = 7, respectively; Fig. 7d, e, indicating inconsistency between the latency of seizure on-set and seizure-induced CA3 neuronal degeneration. Regarding that KA did not affect p65 S276 phosphorylation and COX-2 expression in *PLPP/CIN*^*−/−*^ mice (Fig. 7f), our findings indicate that PLPP/CIN may regulate NF2-PAK1-NF-κB-COX-2-PTGES2 signaling pathway, which increase the seizure threshold in response to KA.

## Discussion

**Fig. 8:**
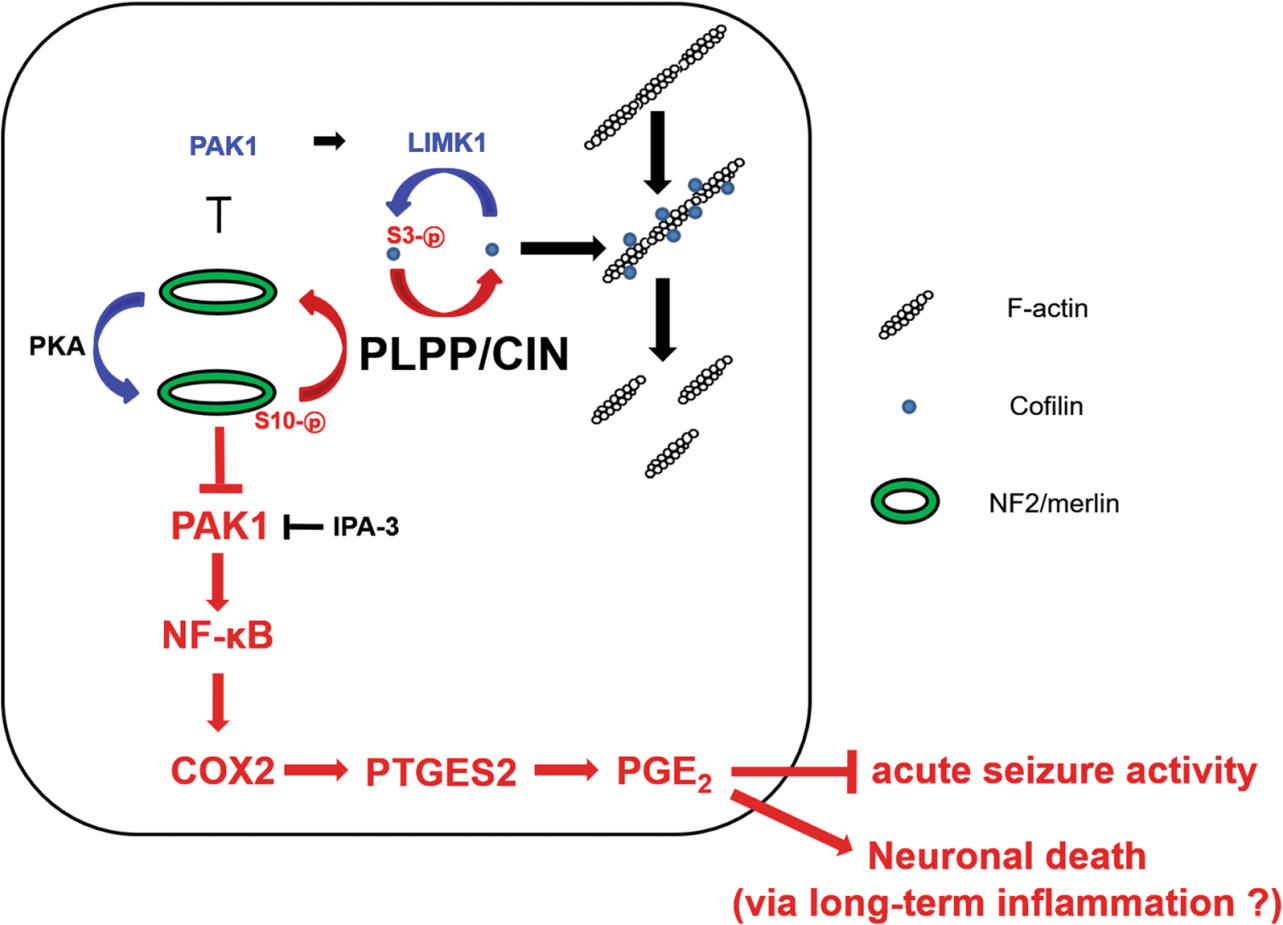
Scheme of the role of PLPP/CIN-mediated NF2 S10 dephosphorylation in response to KA. Following KA treatment, PLPP/CIN-mediated NF2 S10 dephosphorylation may activate PAK1-NF-κB-COX-2-PTGES2 signaling pathway in neurons, which would subsequently increase the latency of seizure on-set and CA3 neuronal damage in response to KA

The major findings in the present study are that PLPP/CIN increased PAK1 S204 autophosphorylation mediated by NF2 S10 dephosphorylation in hippocampal neurons under physiological condition, which facilitated NF-κB activation, COX-2 upregulation and PTGES2 induction following KA treatment. Furthermore, PAK1 inhibition by IPA-3 shortened the latency of seizure on-set, abrogated COX-2 and PTGES2 upregulations and attenuated CA3 neuronal damage induced by KA without affecting seizure intensity. Therefore, our findings suggest that PLPP/CIN may regulate seizure susceptibility (the latency of seizure on-set) and seizure-induced CA3 neuronal degeneration in response to KA through NF2-PAK1-NF-κB-COX-2-PTGES2 signaling pathway (Fig. 8).

Although the shorter latency of seizure on-set is generally thought to represent the higher seizure intensity and to exert its progression, many literatures describe the inconsistency between seizure threshold and intensity. Deletion of dopamine β-hydroxylase or norepinephrine transporter deletion decreases seizure threshold and its seizure duration [56, 57]. In addition, lack of inducible heat shock protein 70, acid-sensing ion channel 1a or galanin receptor 2 also distinctly affect seizure susceptibility and its severity [58–60]. PLPP/CIN overexpression increases the latency of seizure on-set and seizure intensity, while its ablation shortens the latency of seizure on-set and abrogates seizure progression in response to KA [7–11]. Therefore, it is likely that the PLPP/CIN-mediated signaling pathways may distinctly modulate ictogenesis and seizure progression, while underlying mechanisms are largely unknown.

Recently, we have reported that PLPP/CIN selectively dephosphorylates NF2 S10 site, which increases seizure intensity and its progression in response to KA by regulating F-actin stability, NMDAR-PSD95 co-assembly and NF2-mediated Mdm2 degradation [7]. In the present study, we found that PLPP/CIN-mediated NF2 S10 dephosphorylation increased PAK1 S204 phosphorylation under physiological condition, which were enhanced by KA treatment. Furthermore, PAK1 inhibition by IPA-3 decreased seizure threshold (shortened the latency of seizure on-set) in response to KA without affecting seizure duration and its progression. Considering NF2-mediated suppression of PAK1 activity by preventing the Cdc42/Rac1 binding to N-terminal regulatory domain of PAK1 [23], our findings indicate that PLPP/CIN-mediated NF2 S10 dephosphorylation may facilitate PAK1 activation by enhancing its S204 phosphorylation under physiological and post-KA conditions, which would increase the latency of seizure on-set in response to KA. Indeed, PAK1 mutation is relevant to neurodevelopmental disorder showing epileptic symptoms [17, 61, 62]. In contrast to these reports, the higher PAK1 S199/S204 phosphorylations are relevant to the seizure susceptibility in fragile X mental retardation 1 (*Fmr1*) KO mice (a Fragile X syndrome model), since FRAX486 (a PAKs inhibitor) reduces audiogenic seizure activity in this model [63, 64]. However, the seizure susceptibility of *Fmr1* KO mice in response to KA is similar to that of WT mice [65]. Unlike the case of *Fmr1* KO mice, the present study reveals that PLPP/CIN-mediated PAK1 S204 phosphorylation may increase the latency of seizure on-set, and that IPA-3 increased seizure susceptibility in response to KA without seizure intensity and its progression, suggesting that PLPP/CIN-NF2-PAK1 signaling pathway may modulate seizure susceptibility in response to KA.

It has been well known that NF2 inhibits PAK1-mediated NF-κB transactivation [36, 37, 40]. Interestingly, NF-κB inhibition reduces seizure threshold in response to KA [39]. In the present study, PLPP/CIN-mediated PAK1 S204 phosphorylation increased NF-κB p65 S276 phosphorylation under physiological condition and more enhanced it following KA treatment. In addition, IPA-3 abolished p65 S276 phosphorylation and inductions of COX-2 and PTGES2 in CA3 neurons concomitant with the increased seizure susceptibility following KA treatment. Since NF-κB is an upstream transcription factor of COX-2 [54, 55], our findings indicate that PLPP/CIN-mediated PAK1 S204 phosphorylation may delay seizure on-set in response to KA by activating NF-κB-mediated COX-2 upregulation, which would facilitate PTGES2 induction. Indeed, the genetic deletion and inhibition of COX-2 or PTGES2 exacerbate seizure activity by reducing GABAergic inhibitions as well as activating glutamatergic excitation [50–52]. Furthermore, PGE_2_ possesses anti-convulsive properties in acute seizure models [47, 49, 66]. COX-2 and PGE_2_ also mediates neuroinflammation leading to neuronal damage [42]. KA immediately results in COX-2 upregulation in CA3 neurons in the acute phase (30 min after injection), and later increases it in non-neuronal cells, such as astrocytes and endothelial cells (8 h after injection). However, the early neuronal COX-2 induction does not play a causative role in neuronal damage and inflammation, but leads to the later non-neuronal COX-2 upregulation to promote them [53, 67–69]. Compatible with these reports, the present data demonstrate that PLPP/CIN overexpression aggravated CA3 neuronal damage at 3 days after KA injection. Furthermore, IPA-3 pretreatment ameliorated CA3 neuronal degeneration induced by KA in WT and *PLPP/CIN*^*Tg*^ mice, while it shortened the latency of KA-induced seizure on-set without altering seizure intensity and behavioral seizure severity in both strains. This inconsistency between the latency of seizure on-set and CA3 neuronal damage indicates that protective effect of IPA-3 on seizure-induced CA3 neuronal degeneration may be relevant to COX-2-PTGES2-mediated events rather than the degree of seizure activity/duration. Taken together, our findings suggest that PLPP/CIN-mediated regulation of NF2-PAK1-NF-κB-COX-2-PTGES2 may decrease seizure susceptibility to KA, but enhance the subsequent inflammatory responses leading to seizure-induced CA3 neuronal death.

On the other hand, KA did not affect PAK1 S204 phosphorylation in *PLPP/CIN*^*−/−*^ mice, although it increased NF2 S10 phosphorylation level. Considering weak seizure activity in *PLPP/CIN*^*−/−*^ mice in response to KA, it is plausible that the low seizure intensity may lead to the dissociation between NF2 dephosphorylation and PAK1 phosphorylation. Adversely, the increased NF2 S10 phosphorylation induced by KA may be insufficient to induce PAK1 S204 phosphorylation or be involved in F-actin stability rather than PAK1 autophosphorylation [7] in *PLPP/CIN*^*−/−*^ mice. However, it is not excluded the possibility that other signaling molecules would be involved in the regulation of KA-induced PAK1 autophosphorylation, independent of NF2. Further studies are needed to elucidate NF2-independent regulation of PAK1 autophosphorylation in *PLPP/CIN*^*−/−*^ mice.

## Conclusion

The present data we demonstrate for the first time that PLPP/CIN-mediated NF2 S10 dephosphorylation may reduce seizure susceptibility and augment CA3 neuronal damage in response to KA by activating PAK1-NF-κB-COX-2-PTGES signaling pathway. Therefore, detailed elucidation of this pathway will be of great importance in understanding the underlying mechanisms of ictogenesis and seizure-induced neuronal death.

## Supplementary Information

Additional file 1: Figure S1. Full-length gel images of Western blots in Fig. 1. Figure S2. Full-length gel images of Western blots in Fig. 2. Figure S3. Full-length gel images of Western blots in Fig. 3. Figure S4. Full-length gel images of Western blots in Fig. 5. Figure S5. Full-length gel images of Western blots in Fig. 7.

## Data Availability

All data generated or analyzed during this study are included in this published article and its additional files.
