# Supplementary material for: PLPP/CIN-mediated NF2 S10 dephosphorylation distinctly regulates kainate-induced seizure susceptibility and neuronal death through PAK1-NF-κB-COX-2-PTGES2 signaling pathway
Source: J Neuroinflammation. 2023 Apr 28;20:99. doi: 10.1186/s12974-023-02788-9 (PMC10141957; doi:10.1186/s12974-023-02788-9)
Supplement: Supplementary file 1 — Additional file 1: Figure S1. Full-length gel images of Western blots in Fig. 1. Figure S2. Full-length gel images of Western blots in Fig. 2. Figure S3. Full-length gel images of Western blots in Fig. 3. Figure S4. Full-length gel images of Western blots in Fig. 5. Figure S5. Full-length gel images of Western blots in Fig. 7. [file 12974_2023_2788_MOESM1_ESM.pdf]

PLPP/CIN-mediated NF2 S10 dephosphorylation distinctly regulates kainate-induced seizure susceptibility and neuronal death through PAK1-NF-κB-COX-2-PTGES2 signaling pathway

Ji-Eun Kim[1], Duk-Shin Lee[1], Tae-Hyun Kim[1], Hana Park[1], Min-Ju Kim[1] and Tae-Cheon Kang[1*]

[1]Department of Anatomy and Neurobiology, Institute of Epilepsy Research, College of Medicine, Hallym University, Chuncheon 24252, South Korea

* Correspondence to: T.-C Kang, Department of Anatomy and Neurobiology, College of Medicine, Hallym University, Chuncheon, Kangwon-Do 24252, South Korea; Tel: +82-33-248-2524; Fax: +82-33-248-2525; E-mail: tckang@hallym.ac.kr

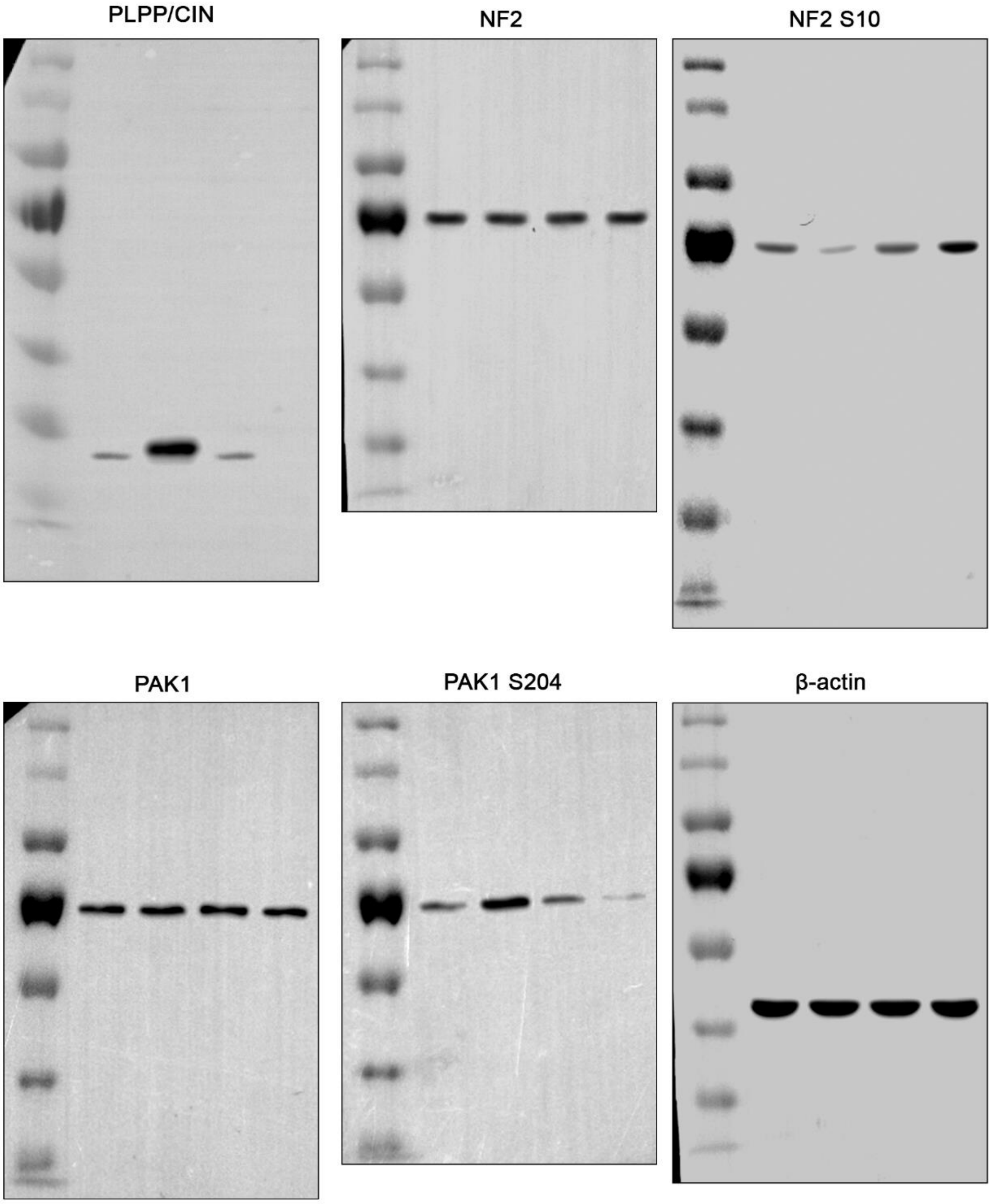

Additional file 1 : Figure S1. Full-length gel images of Western blots in Figure 1.

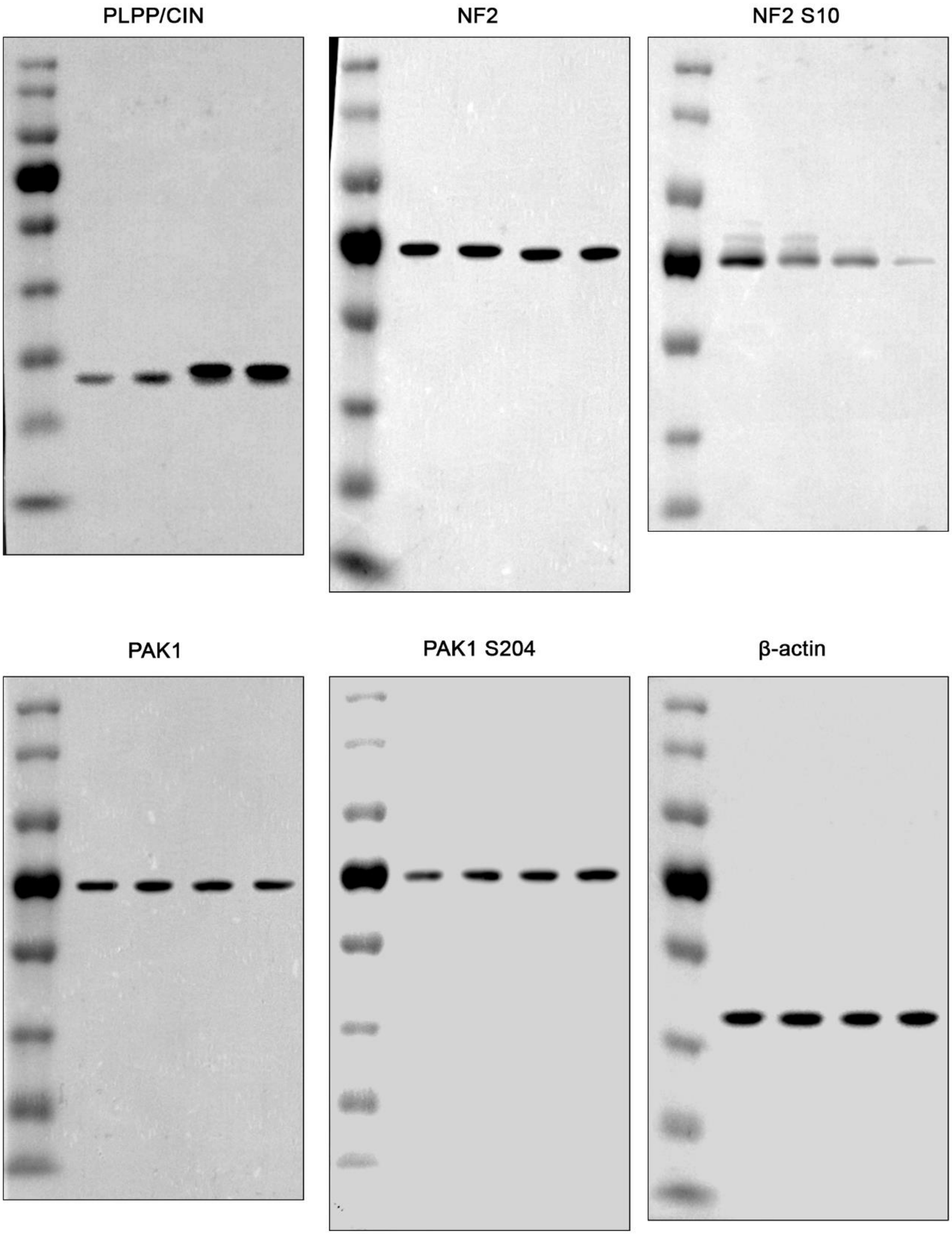

Additional file 1: Figure S2. Full-length gel images of Western blots in Figure 2.

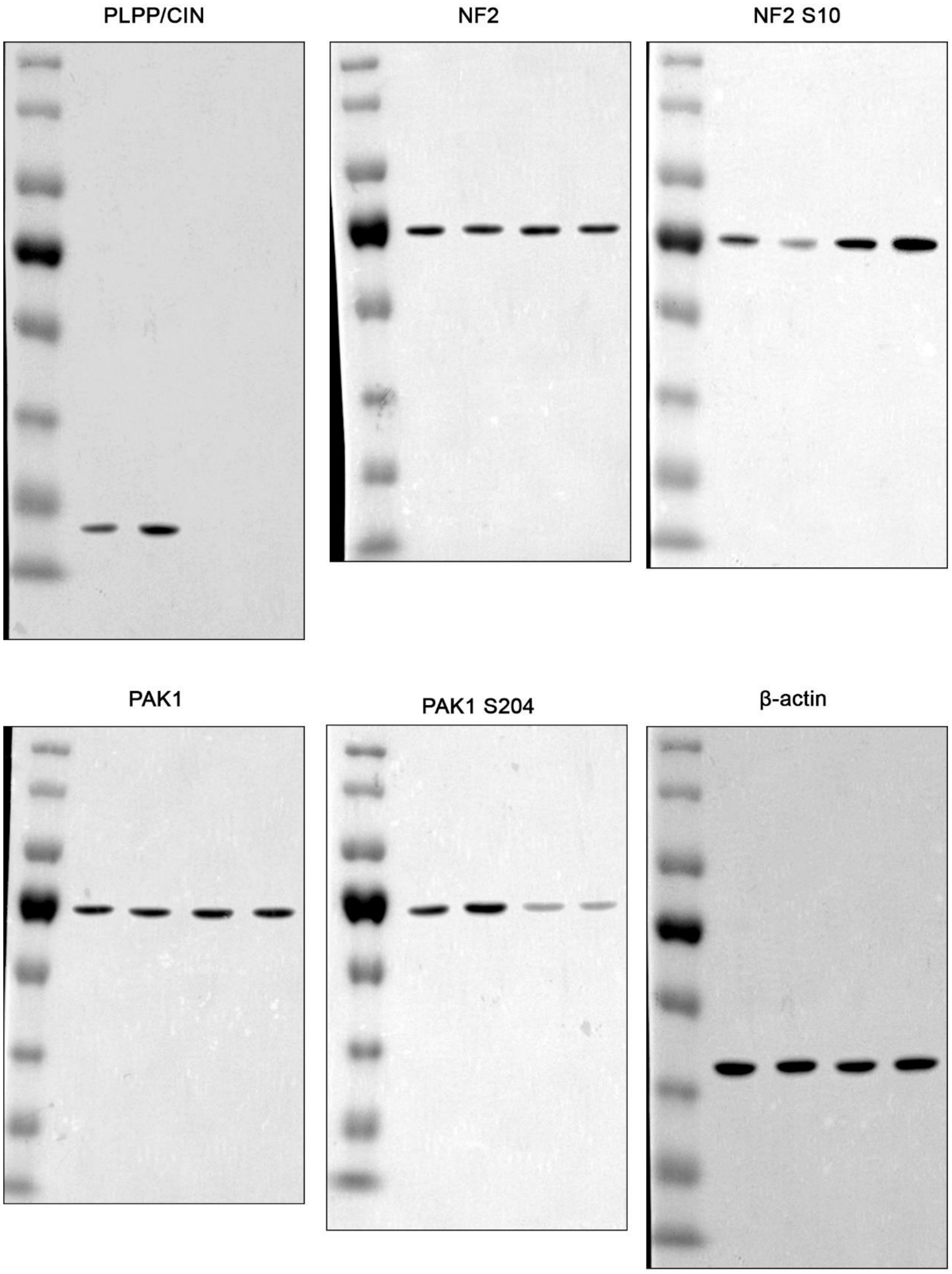

Additional file 1: Figure S3. Full-length gel images of Western blots in Figure 3.

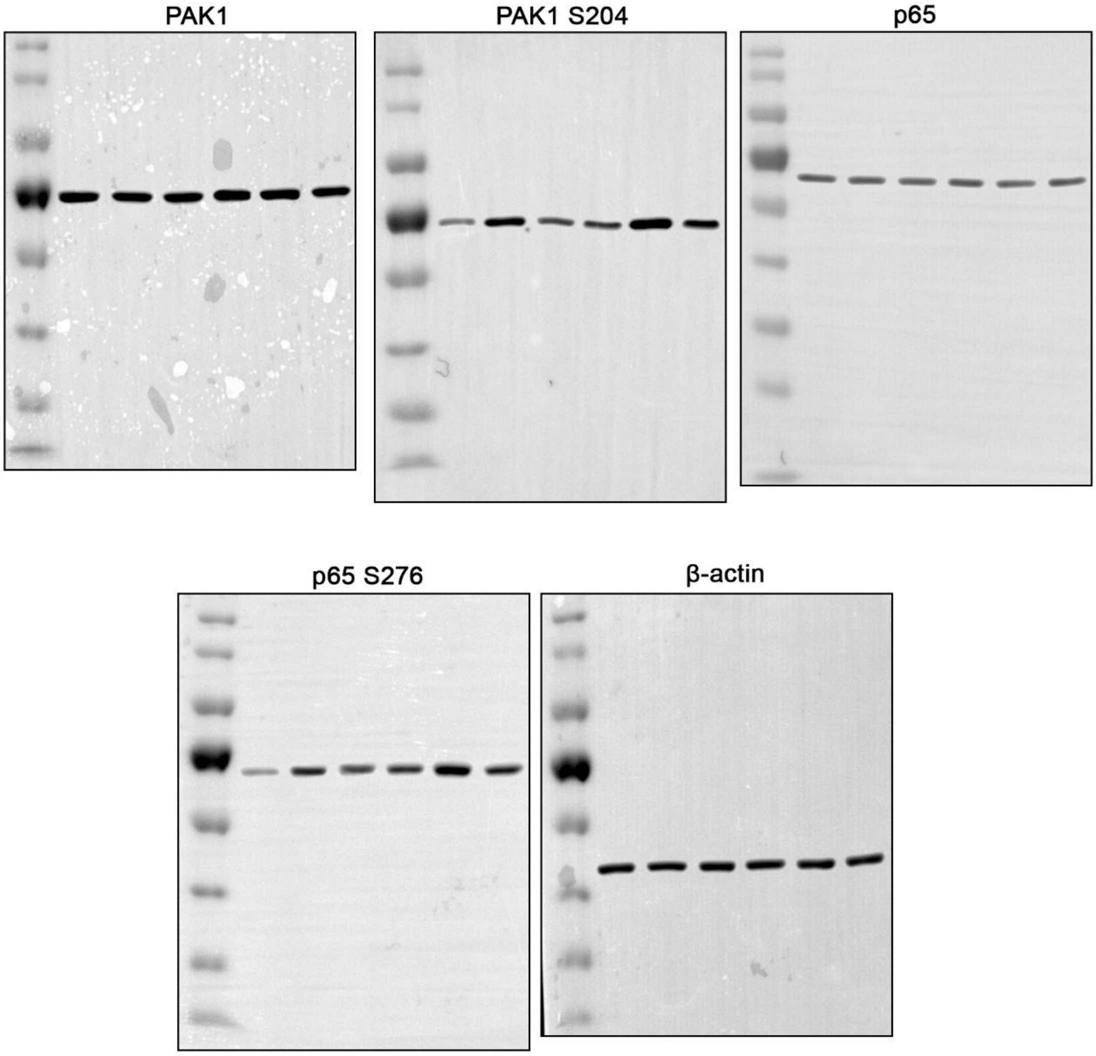

Additional file 1: Figure S4. Full-length gel images of Western blots in Figure 5.

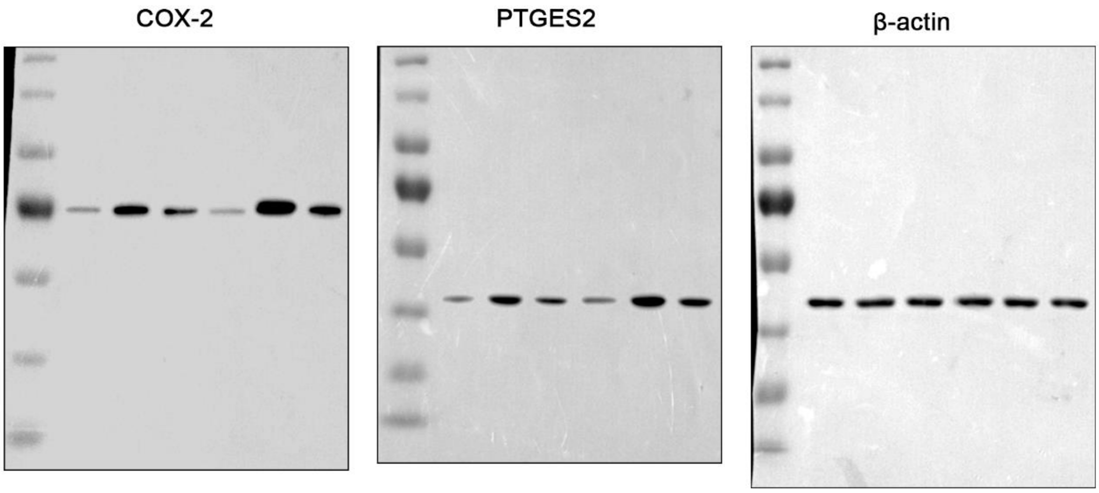

Additional file 1: Figure S5. Full-length gel images of Western blots in Figure 7.
